# Prehospital Delay and Its Associated Factors in Sudanese Patients Presenting With Acute Appendicitis at a Teaching Hospital

**DOI:** 10.7759/cureus.23036

**Published:** 2022-03-10

**Authors:** Qasem Alyhari, Faisal Ahmed, Mohamed Nasreldin, Hossein-Ali Nikbakht, Ahmad Alamin, Saleh Al-Wageeh, Saif Ghabisha, Ebrahim Al-Shami, Fawaz Mohammed

**Affiliations:** 1 Department of General Surgery, School of Medicine, Ibb University of Medical Science, Ibb, YEM; 2 Urology Research Center, Al-Thora General Hospital, Department of Urology, School of Medicine, Ibb University of Medical Science, Ibb, YEM; 3 Department of General Surgery, Faculty of Medicine, Gezira University, Gezira, SDN; 4 Social Determinants of Health Research Center, Health Research Institute, Babol University of Medical Sciences, Babol, IRN; 5 Department of Orthopedics, School of Medicine, Ibb University of Medical Science, Ibb, YEM

**Keywords:** prehospital, complications, diagnosis, late presentation, acute appendicitis

## Abstract

Background

Delayed prehospital presentation of acute appendicitis may increase the risk of perforation and other complications. This study investigated the prevalence of prehospital delay in the presentation of acute appendicitis, clinical features, and outcomes in Sudanese patients.

Method

A retrospective study conducted from January 2017 to December 2020 in a teaching hospital affiliated with Gezira University enrolled 191 patients with prehospital delay presentation of acute appendicitis (at least 48 hours from symptom onset). Patient characteristics, causative factors, primary treatment, and complication rate were gathered and analyzed.

Result

The mean age of the patients was 36.55 ± 16.3 years (range: 15-78 years), with 122 (64%) males and 69 (36%) females. Most cases of prehospital delay were misdiagnosed firstly as other diseases (n = 124, 65%). The physicians made misdiagnosis of acute appendicitis in 65 (53%) patients. Age less than 30 years, male gender, living in rural areas, and lower educational level are associated with a high incidence of prehospital delay presentation of acute appendicitis (p < 0.05). Most cases have appendicular mass (46%, p < 0.001). Wound infection was the most common postoperative complication (7.85%, p < 0.001).

Conclusion

The high incidence rate of prehospital delay presentation of acute appendicitis is associated with patients' age ofless than 30 years, male gender, living in a rural area, and lower educational level. With the high rate of misdiagnosed acute appendicitis, it is essential to increase the knowledge about the signs and symptoms of appendicitis among our physicians and health practitioners.

## Introduction

Acute appendicitis is one of the most common surgical emergency cases [1]. During admissions, acute appendicitis is misdiagnosed in 3.8%-15% of all children cases and 5.95%-23.5% of all cases [2]. Its postoperative complication and morbidity rates remain high, about 29% and 18%, respectively [3]. Prehospital delay of acute appendicitis is defined as the time interval from when a patient with a history of acute appendicitis comes after at least 48 hours of symptom onset [2].

Many research findings have found a correlation between the time interval and the risk of appendicitis perforation; a long wait before operation results in complicated appendicitis and, as a result, high postoperative morbidity [4-7]. Prehospital delay may have played a more significant role in the progression of appendicitis and future postoperative complications [3,5]. According to Li et al., delayed prehospital presentation for acute appendicitis was associated with older age, residing alone, lack of knowledge about the disease, low social support, negative characteristics of mental well-being such as introvert personality and poor coping style, low severity of the pain, and symptoms occurring during work hours [3]. This study investigated the prevalence of late prehospital presentation of acute appendicitis, clinical features, and outcomes in Sudanese patients.

## Materials and methods

Study design

A retrospective study was conducted between January 2017 and December 2020 at the Wad Medani Teaching Hospital, which was affiliated with Gezira University, Sudan. Of 758 patients with acute appendicitis, 191 (25%) patients with a late presentation of acute appendicitis were collected and enrolled in this study. The late prehospital presentation of acute appendicitis was defined as a period from symptom onset to the entrance to the emergency department that was greater than 48 hours [3,8,9]. The inclusion criteria were any patient presented to Wad Medani Teaching Hospital with a late prehospital presentation of acute appendicitis in the study period. The exclusion criteria include other causes of the acute surgical abdomen, patients diagnosed or operated on by another hospital, and patients who could not provide sufficient information.

Data collection

Data were collected from hospital records of patients managed with late prehospital presentation. It includes personal data such as age, sex, and educational level, subject disease history, case of late prehospital presentation, management, and postoperative complications.

Statistical analysis

Mean and standard deviation were used for quantitative variables, and frequency and percentage were used for qualitative variables. The chi-square test was used to determine the relationship between nominal and categorical variables in terms of the number of causative factors of delayed prehospital presentation of acute appendicitis. A p-value of 0. 05 or less was considered statistically significant. All statistical analyses were done using the STATA software version 13 (StataCorp LLC, College Station, Texas, USA).

## Results

Out of the 758 patients diagnosed with acute appendicitis, 191 (25%) patients presented with prehospital delay presentation. About 122 (64%) patients were male, and 69 (36%) were female. The mean age of the patients was 36.55 ± 16.3 years (range: 15-78 years). The higher incidence rate of late prehospital presentation of acute appendicitis was seen in the age group of less than 30 years, which was 47.6%. Regarding educational level, 63 (33%) patients were found to be illiterate, 51 (26.7%) completed primary school, and 44 (23%) completed high school. About 157 (82%) patients live in rural areas, and 34 (18%) live in urban areas. Only 8% of the patients had comorbidities such as diabetes mellitus and hypertension (Table 1).

**Table 1:** Characteristics of patients with prehospital delay of acute appendicitis (N = 191)

Variables	Subgroups	N (%)
Age (year)		36.55 ± 16.3 (range: 15–78 years)
Age group	Less than 30 years	91 (47.6%)
	30–45 years	52 (27.2%)
	45–60 years	28 (14.7%)
	More than 60 years	20 (10.5%)
Gender	Male	122 (64%)
	Female	69 (36%)
Educational level	Illiterate	63 (33%)
	Completed primary school	51 (26.7%)
	Completed high school	44 (23%)
Place of residence	Rural area	157 (82%)
	Urban area	34 (18%)
Comorbidity	Yes	16 (8%)
	No	175 (92%)
Causes of delayed presentation	Misdiagnosed as other diseases	124 (65%)
	Treated traditionally	31 (16%)
	Ignorance	36 (19%)

The causes of delayed prehospital presentation to our surgery department are as follows: 124 (65%) patients were misdiagnosed with other diseases, 31 (16%) patients were treated traditionally, and 36 (19%) patients had ignorance about treating acute abdominal pain. The main misdiagnosed items were malaria, gastroenteritis, and typhoid fever in 23%, 22%, and 21%, respectively. Physicians misdiagnosed acute appendicitis in 65 (53%) patients, 55 (44%) patients by medical assistants and nurses, and four (3%) patients by the pharmacist (Table 2).

**Table 2:** Characteristics of misdiagnosed cases (n = 124, 65%)

Variables	Subgroups	N (%)
Visited by	General physician	65 (53%)
	Medical assistant or nurse	55 (44%)
	Pharmacist	4 (3%)
Misdiagnosis	Malaria	29 (23.4%)
	Typhoid	26 (21%)
	Inflammatory bowel syndrome	7 (5.6%)
	Gastroenteritis	28 (22.6%)
	Pelvic inflammatory disease	17 (13.7%)
	Urinary tract infection	15 (12.1%)
	Renal stone	1 (0.8%)
	Gallstone	1 (0.8%)

The final diagnoses were as follows: appendicular mass in 88 (46%) patients, peritonitis due to perforated appendix in 48 (25%) patients, appendicular abscess in 15 (8%) patients, and acute appendicitis in 40 (21%) patients. According to management, 41 (22%) patients underwent appendectomy, 18 (9%) patients underwent drainage of abscess, and 53 (28%) patients underwent abdominal laparotomy. The majority of patients with appendicular mass were treated conservatively (n = 78, 89%), 11% failed the conservative management, 4.5% developed an appendicular abscess and underwent drainage of the abscess, and 6.5% developed peritonitis and underwent laparotomy.

The total hospital stay was between three and five days in 105 (55%) patients, more than five days in 52 (27%) patients, and three days in 34 (18%) patients. There were a total of 18 (9.4%) postoperative complications, including wound infection in 15 (7.85%) patients, which was the most typical complication, burst abdomen in two (1.04%) patients, and death in one (0.5%) patient (Table 3).

**Table 3:** Characteristics of patients during hospital admission

Variables	Subgroup	N (%)
Diagnosis	Appendicular mass	88 (46%)
	Peritonitis due to a perforated appendix	48 (25%)
	Appendicular abscess	15 (8%)
	Acute appendicitis	40 (21%)
Management	Appendectomy	41 (21.5%)
	Drainage of abscess	18 (9.4%)
	Laparotomy	53 (27.7%)
	Conservative management	79 (41.4%)
Postoperative complications	Wound infection	15 (7.85%)
	Burst abdomen	2 (1.04%)
	Death	1 (0.5%)
Hospital stays	Three days	34 (18%)
	3–5 days	105 (55%)
	More than five days	52 (27%)

Relationship between the causative factors of delayed prehospital presentation of appendicitis

The chi-square test showed that age less than 30 years, male gender, living in a rural area, and lower educational level are associated with a high incidence of delayed prehospital presentation of acute appendicitis (p < 0.05).

Most cases of delayed prehospital presentation of acute appendicitis were diagnosed as other diseases (65%, p < 0.001). Additionally, most cases with a delayed prehospital presentation of acute appendicitis have appendicular mass (46%, p < 0.001), and most patients were misdiagnosed by the general physician (34%, p < 0.001).

Regarding postoperative complications, wound infection was the most common postoperative complication (7.85%, p < 0.001) (Table 4).

**Table 4:** Investigation of the causative factors of delayed hospital presentation of appendicitis (N = 191)

Variables		N (%)	p-value
Age group	Less than 30 years	91 (47.6%)	<0.001
	30–45 years	52 (27.2%)	
	45–60 years	28 (14.7%)	
	More than 60 years	20 (10.5%)	
Gender	Male	122 (64%)	0.002
	Female	69 (36%)	
Place	Rural area	157 (82%)	<0.001
	Urban area	34 (18%)	
Educational level	Illiterate	63 (33%)	0.256
	Completed high school	51 (26.7%)	
	Completed primary school	44 (23%)	
Causes of delayed presentation	Misdiagnosed with other diseases	124 (65%)	<0.001
	Treated traditionally	31 (16%)	
	Ignorance	36 (19%)	
Diagnosis	Appendicular mass	88 (46%)	<0.001
	Peritonitis	48 (25%)	
	Appendicular abscess	15 (8%)	
	Acute appendicitis	40 (21%)	
Misdiagnosed by	Others	67 (35.2%)	<0.001
	General physician	65 (34%)	
	Medical assistant or nurse	55 (28.7%)	
	Pharmacist	4 (2.1%)	
Management	Appendectomy	41 (21.5%)	<0.001
	Drainage of abscess	18 (9.4%)	
	Laparotomy	53 (27.7%)	
	Conservative management	79 (41.4%)	
Postoperative complications	Wound infection	15 (7.85%)	<0.001
	Burst abdomen	2 (1.04%)	
	Death	1 (0.5%)	
Hospital stays	Three days	34 (18%)	<0.001
	3–5 days	105 (55%)	
	More than five days	52 (27%)	

## Discussion

In this study of 758 patients with acute appendicitis, 191 (25%) cases presented with delayed prehospital presentation (after 48 hours of symptom onset). Our result showed that age less than 30 years, male gender, rural area patients, and lower educational level are associated with a high incidence of delayed prehospital presentation of appendicitis. Additionally, most cases have appendicular mass (46%), and wound infection was the most common postoperative complication.

Previous studies focused on the relationship between the system time (length of time from the entrance of the emergency department to the surgery room) and the role it played in the progression of acute appendicitis; this study considered the role of patient time (period from symptom onset to the entrance in the emergency department) [10]. It was mentioned that after the first 36 hours from the onset of appendicitis symptoms, the average appendiceal perforation rate is between 16% and 36%, with a 5% risk of perforation for each subsequent 12-hour period [11,12].

In our study, 25% of the cases came to our hospital after 48 hours. Li et al. reported a higher number of prehospital delays in Chinese patients, about 57.5% [3]. In other studies, Flum et al. and Flum et al. reported that the prehospital delay in appendicitis was 15.3% [2,13]. The different rate between studies may be due to various sociodemographic characteristics, clinical factors, cognitive factors, and psychosocial factors. Prehospital delay is heavily influenced by socioeconomic factors, particularly in developing countries such as Sudan.

Our result revealed that delayed prehospital presentation was more in patients under 30 years of age. Our result was similar to the previously published study in Sudan by Doumi and Abdelrahman; the peak incidence of acute appendicitis in this study was around 20-30 years [14]. Another study in Nigeria reported that the highest incidence of acute appendicitis was in ages 11-30 years [15]. In contrast, a recent study in China by Li et al. found that the rate of delayed prehospital presentation of acute appendicitis was significant in older patients (age ≥ 60 years) [3]. Our explanation for this issue is that insurance is not popular in Sudan and the hospital administration process is expensive and time-consuming, which makes younger patients avoid coming to hospitals and treat themself with traditional therapies or visit inexperienced general physicians.

In our study, a high male predominance was observed, and the same ratio was seen in the study of Doumi and Abdelrahman [14]. On the contrary, another study reported no difference between males and females who presented a late prehospital presentation of acute appendicitis [3]. It was mentioned that gender did not affect the postoperative complication rate and was not a predisposing factor for the late prehospital presentation of appendicitis [3,15].

Most prehospitalization delayed patients in our study live in rural areas (82%). Similarly, Paquette et al. showed that patients from rural areas have higher rates of acute appendicitis than patients from urban areas [16]. The high incidence in rural areas may be due to hard access to high hospital centers and high misdiagnosis of acute appendicitis by general physicians.

In our study, the most common causes of delayed prehospital presentation of acute appendicitis were misdiagnosis in medical centers (65%). Of these misdiagnosed patients, 53% were visited by general physicians and 44% by medical assistants or nurses, and 3% took drugs from pharmacists without consultation. Furthermore, approximately 19% of our patients were unaware of abdominal pain treatment and did not seek medical advice due to poor education, and 16% of the cases were traditionally treated. According to Asad et al., 23% of complicated appendicitis presentation was delayed due to missed diagnosis by physicians, 30% were delayed due to missed diagnosis by non-doctors, 23.08% were delayed due to conservative management by surgeons, and 23% were delayed due to self-medication at home [17]. In comparison, Mutwali et al. reported that 50.2% of misdiagnosed appendicitis were visited by a doctor [18]. Additionally, Osifo et al. reported that about 60% of appendicitis cases had a wrong diagnosis [19]. All these studies showed that most cases with late prehospital presentation had already sought medical advice before going to the surgery department. As a result, more medical education on appendicitis signs should be conducted among general physicians to avoid this problem.

Our result showed that the misdiagnosed cases were distributed as follows: 23.4%, malaria; 21%, typhoid; 22.6%, gastroenteritis; 13.7%, pelvic inflammatory disorder; 12.1%, urinary tract infections; and 5.6%, inflammatory bowel disease. Similarly, Doumi and Abdelrahman reported that among 101 cases of acute appendicitis, 71 cases had received antimalarial drugs, 14 had received anti-amoebiasis, eight cases were treated for colitis, and the rest were treated as a pelvic inflammatory disease [14]. In Hong Kong, Chung et al. reported that 64.5% of cases were diagnosed as nonspecific abdominal pain, 32.2% were diagnosed as gastroenteritis, and 3.2% were diagnosed as urinary tract infection [20]. When we see most diseases in misdiagnosed patients as signs and symptoms of acute appendicitis, proper history and examination are essential for diagnosis.

Patients presented at our hospital as follows: 46% with appendicular mass, 25% with peritonitis due to a perforated appendix and underwent laparotomies, 8% with appendicular abscess and underwent drainage of abscess, and 21% with acute appendicitis and underwent appendectomies. In comparison with a study by Doumi and Abdelrahman, nearly 34% of cases had appendicular mass, 5% of cases had peritonitis due to a perforated appendix and underwent laparotomy, 4% of cases had an appendicular abscess and underwent drainage of abscess, and 57% of cases had acute appendicitis and underwent appendectomy [14]. In another study by Chung et al., the authors reported that 60% of cases had acute appendicitis, 17% had perforated appendix, and 23% had appendicular abscess [20]. A recent study by Khan et al. investigated complicated appendicitis in toddlers and found perforated appendicitis with a localized abscess in 68 patients, gangrenous appendicitis in four patients, generalized peritonitis in 24 patients, and appendicular mass in 12 patients [21]. Our result was in some agreement with the previously mentioned researchers.

According to the cases of appendicular mass, 89% were treated conservatively, but 11% failed the conservative management as 4.5% developed an appendicular abscess and underwent drainage of the abscess and 6.5% developed peritonitis and underwent laparotomy. In the article by Mutwali et al., 84% of appendicular mass were treated conservatively, and 16% were treated surgically [18]. In contrast, Doumi and Abdelrahman reported that all cases with appendicular mass were treated conservatively [14]. This study's high rate of peritonitis may be due to more prehospital time spent in our patients that lead to more peritonitis.

The total postoperative complications were 18 (9.4%), which included wound infection in 15 (7.85%) patients, the most common complication. Additionally, two (1.04%) patients had burst abdomen, and one (0.5%) patient died. In 113 cases of appendicitis, Ayoade et al. found that the most typical postoperative complication was high-grade fever in 16 (14.2%) cases, followed by wound infection in 12 (10.6%) and death in one (0.9%) [22]. A systematic review showed that 12-24 hours of delayed operation timing was not related to the risk of complex appendicitis; however, after 48 hours, surgical site infection and 30-day adverse events increased [23].

The time spent in the hospital was as follows: 55% stayed for three to five days, 27% stayed more than five days, and 18% were discharged within three days. Mutwali et al. mentioned that the mean hospital stay for the conservative treatment group was 5.4 ± 2.65 days and that for the surgical treatment group was 7.13 ± 3.91 days [18]. Additionally, Salati et al. found that the mean hospital stay for patients with perforated appendicitis was 7.7 days [24].

The published article on 151 patients showed that the referral region as remote, abscess, perforation, preoperative hospital delay, and referral duration were the main factors affecting the length of hospital stay for patients with acute appendicitis [25]. In another report, among 300 patients operated on for acute appendicitis, 36 (12%) developed unfavorable results. Although one death because of sepsis was recorded, wound infection was the major postoperative disorder. The authors reported that living outside of the hospital setting area, long duration of illness before arrival at the hospital, more than three days of hospital stay, and mass in the right lower quadrant were risk factors for unfavorable outcomes. The time spent in the hospital was as follows: 67.3% stayed less than three days, 31.3% stayed for three to seven days, and 1.3% stayed more than seven days [26]. In some agreement with previous reports, we reported that age less than 30 years, male gender, living in a rural area, and lower educational level are associated with a high incidence of delayed prehospital presentation of appendicitis.

There are several limitations to our study. The present study was a retrospective study based on experiences from a single unit. Therefore, it is not easy to extrapolate the findings to the entire community. Another limitation was the small sample size with little robust statistical analysis. Additional research with a more extensive prospective cohort study is required to validate our findings. Furthermore, this study did not include additional details such as blood test results, symptoms at admission, and radiological images, which was an unavoidable limitation.

## Conclusions

The high incidence rate of late prehospital presentation of acute appendicitis is associated with age less than 30 years, male gender, living in rural areas, and lower educational level. With the high rate of misdiagnosed acute appendicitis, it is essential to increase the knowledge about the signs and symptoms of appendicitis among our physicians and health practitioners.
